# HA117 endows HL60 cells with a stem-like signature by inhibiting the degradation of DNMT1 via its ability to down-regulate expression of the GGL domain of RGS6

**DOI:** 10.1371/journal.pone.0180142

**Published:** 2017-06-30

**Authors:** Shuangshuang Li, Xianqing Jin, Huan Wu, Yi Wang, Xiaoqing Li, Yuxia Guo, Shaoyan Liang

**Affiliations:** 1 Department of Gastrointestinal Surgery and Neonatal Surgery, Children's Hospital of Chongqing Medical University, Ministry of Education Key Laboratory of Child Development and Disorders, Chongqing, China; 2 China International Science and Technology Cooperation base of Child Development and Critical Disorders, Chongqing Medical University, Chongqing, China; 3 Chongqing Key Laboratory of Pediatrics, Chongqing Medical University, Chongqing, China; 4 Department of Pediatrics, Chongqing Medical University, Chongqing, China; University of South Alabama Mitchell Cancer Institute, UNITED STATES

## Abstract

All-trans retinoic acid (ATRA) induces complete remission in almost all patients with acute promyelocytic leukemia (APL) via its ability to induce the in vivo differentiation of APL blasts. However, prolonged ATRA treatment can result in drug resistance. In previous studies, we generated a multi-drug-resistant HL60/ATRA cell line and found it to contain a new drug resistance-related gene segment, HA117. In this study, we demonstrate that ATRA induces multi-drug-resistant subpopulations of HL60 cells with a putative stem-like signature by up-regulating the expression of the new gene segment HA117. Western blot analysis and quantitative real-time PCR demonstrated that HA117 causes alternative splicing of regulator of G-protein signaling 6 (RGS6) and down-regulation of the expression of the GGL domain of RGS6, which plays an important role in DNA methyltransferase 1 (DNMT1) degradation. Moreover, DNMT1 expression was increased in multi-drug resistance HL60/ATRA cells. Knockdown of HA117 restored expression of the GGL domain and blocked DNMT1 expression. Moreover, resistant cells displayed a putative stem-like signature with increased expression of cancer steam cell markers CD133 and CD123. The stem cell marker, Nanog, was significantly up-regulated. In conclusion, our study shows that HA117 potentially promotes the stem-like signature of the HL60/ATRA cell line by inhibiting by the ubiquitination and degradation of DNMT1 and by down-regulating the expression of the GGL domain of RGS6. These results throw light on the cellular events associated with the ATRA-induced multi-drug resistance phenotype in acute leukemia.

## Introduction

Complete remission is induced by all-trans retinoic acid (ATRA) in almost all patients with acute promyelocytic leukemia (APL) by inducing the differentiation of APL blasts *in vivo*. However, prolonged ATRA treatment can cause drug resistance[1]. Better understanding of the molecular basis of ATRA-induced drug resistance is therefore warranted to exploit the markers and mechanisms underlying this drug-resistant phenotype. Previously, we used ATRA to select drug-resistant HL60 cells, which led to the generation of the multi-drug-resistant cell line, HL-60/ATRA. Suppression subtractive hybridization[2] and microarray analysis of differentially expressed sequences HL-60/ATRA cells enabled us identify a highly expressed sequence, which we refer to as HA117 (GenBank accession number: CB214920)[3]. Bioinformatics analysis of human genomic sequences identified the human gene fragment encoding HA117. The gene is located on the reverse strand of chromosome 14q24.2 in an intergenic region between Regulator of G-protein signaling 6 (RGS6) and Double PHD Fingers Family, Member 3 (DPF3).

RGS6 belongs to the RGS protein family, whose members act as GTPase-activating proteins for Gα subunits to negatively regulate heterotrimeric G-protein signaling [4–6]. RGS6 is distinguished from other members of the RGS family by the presence of GGL and DEP domains in addition to the RGS domain[7]. Multiple splice variants of RGS6 have a long (6L) or short (6S) complete or incomplete GGL domain and N terminus, and diverse C-terminal domains. Moreover, the GGL domain interacts with DNMT1 in a DMAP1-dependent manner [8]. Other experiments reveal that RGS6 acts as a scaffold protein for both DNMT1 and Tip60, and is needed for Tip60-mediated acetylation of DNMT1 and its subsequent ubiquitination and degradation [9].

DNA methylation is among the best studied epigenetic modifications[10] and preserves cellular memory throughout repeated cell divisions[11]. DNMT1 is crucial for the maintenance of hematopoietic stem/progenitor cells [12], epidermal progenitor cells[13], mesenchymal stem cells [14], and leukemia stem cells[15].

Here, we used wild-type HL60 cells and drug-resistant HL60/ATRA cells to show that HA117 promotes the characteristic stem-like signature of these cells by inhibiting by the ubiquitination and degradation of DNMT1 via its ability to down-regulate the GGL domain of RGS6.

## Methods

### Predicting the HA117-RGS6 interaction using LncTar

LncTar from the LncTar package (http://www.cuilab.cn/lnctar) [16] was used to identify potential RNA-RNA interactions and binding sites between HA117 and RGS6. RNA sequences and corresponding annotation data for HA117 and splice variants of RGS6 were retrieved from the NCBI database. All sequences were saved in text files format. LncTar was used to predict interactions between RNAs for HA117 and RGS6 alternatively spliced transcript variants using the command line, perl LncTar.pl -p 1 -l HA117.txt -m RGS6.txt -d -0.05 -s T–o output.txt.

### Cell lines

The HL60 and HL60/ATRA cell lines were provided by the Oncology Laboratory at Children’s Hospital of Chongqing Medical University. The drug-resistant HL60/ATRA cell line and wild-type HL60 were generation-matched and preserved as a suspension culture in RPMI-1640 medium (Thermo Scientific Inc., MA, USA) supplemented with 10% fetal bovine serum (Thermo), L-glutamine, and antibiotics. Cells were incubated at 37°C in a humidified atmosphere of 5% CO_2_.

### Lentiviral infection

HL60/ATRA cells were seeded (2×10^4^ cells per well) in 96-well plates (Corning Incorporated, NY, USA). HA117 RNAi lentivirus (Genechem Co., Ltd, Shanghai, China) was added to HL60/ATRA cells, or an HA117 overexpression lentivirus (GenePharma Co., Ltd, Shanghai, China) was added to RNAi lentivirus-transfected HL60/ATRA cells, in 100 μl of fresh RPMI-1640 containing 10% FBS and 5 μg/ml polybrene. Twelve hours later, an equal volume of medium was added to each well. Three days later, expression of the GFP gene was observed under a fluorescence microscope and the cells were collected for subsequent culture. Cells transfected with empty lentivirus were used as the control group (dx.doi.org/10.17504/protocols.io.h4jb8un).

### Cell proliferation assay

Cells were harvested and plated at a density of 100 cells per well in a 96-well plate (total volume = 100 μl). Then, 10 μl of Cell Counting Kit-8 (Dojindo Molecular Technologies, Inc., Tokyo, Japan) reagent was added to each well for 4 h at 37°C. The optical density was assessed in an automatic microplate spectrophotometer (Varioskan Flash, Thermo) at the wave length of 450 nm. All experiments were carried out in triplicate independently (10.17504/protocols.io.h4kb8uw).

### Cell migration assay

Cells (5×10^5^) were inoculated onto the filter inserts of a polycarbonate membrane in the upper chamber (8 μm pores) (Corning) of a 12-well plate and cultivated in 400 μl RPMI-1640 supplemented with 1% bovine serum albumin (Thermo). The lower chamber was filled with 600 μl of RPMI-1640 medium supplemented with 10% fetal bovine serum. After incubating for 24 h, non-migrating cells on the upper side of polycarbonate membrane were cleared away with swabs. Migrated cells (on the underside) were fixed with formaldehyde for 10 min and then stained with crystal violet (Beyotime Biotechnology, Jiangsu, China). The stained membranes were then cut and placed on a glass slide, and the number of cells that migrated to the bottom surface was counted under an optical microscope. All assays were carried out in triplicate (dx.doi.org/10.17504/protocols.io.h4ib8ue).

### Drug sensitivity assay (CCK8)

Cells (1×10^5^ cells/ml) were inoculated into 96-well plates. Briefly, cells were treated with vincristine (VCR), doxorubicin (ADM), etoposide phosphate (Vp-16), daunorubicin (DNR), mitomycin C (MMC), or cyclophosphamide (CTX) (Solarbio Science & Technology Co., Ltd, Beijing, China) for 24 h before CCK8 (Dojindo) reagent was added to each well for 4 h at 37°C. Absorbance was then read in a plate reader at 450 nm. Sensitivity to the chemotherapeutic agents was calculated after 24 h by measuring proliferation as described above (10.17504/protocols.io.h4mb8u6).

### Real-time polymerase chain reaction (qPCR)

Total RNA was extracted from cell lines Trizol (Invitrogen Life Technologies), according to the manufacturer’s instructions. Next, the first strand cDNA was synthesized from RNA by reverse transcription using PrimeScript^™^ RT reagent Kit (TaKaRa Bio Group, Dalian, China), according to the manufacturer’s instructions. The primers are listed in Table 1. Real-time PCR was performed using the CXF96 (BioRad, CA, USA) system and a SyberGreen II real-time PCR kit (TaKaRa). The PCR reaction conditions were as follows: denaturation at 95°C for 3 min, followed by 39 cycles at 95°C (30 s), 59°C (30 s) and 72°C (30 s), and a final extension at 72°C for 5 min. All statistical analyses were performed using Microsoft Office Excel (10.17504/protocols.io.h4nb8ve).

**Table 1:** Sequences of the primers used for real-time PCR.

Gene	Forward (F) primer	Reverse (R) primer
HA117	CAGAGTCAGGGACTTCAGCCTTAT	CTGTTTCCTTCTCACTCCCAACCA
RGS domain	TTCTGGCTGGCTGTCCAAG	CCTGGGCGTCTTCAAATGTA
DEP domain	TGCCCATCAGAACAGTCAAG	GGGTCCTCAATGGAAAGGTT
GGL domain	CGCACAGATCGACAGACATT	GCTGGTGTTATCAAAGGGTCA
DNMT1	AGGTGGAGAGTTATGACGAGGC	GGTAGAATGCCTGATGGTCTGC
CD133	CACTACCAAGGACAAGGCGTTC	CAACGCCTCTTTGGTCTCCTTG
CD123	CGGAGAATCTGACCTGCTGGAT	GACACTCGTACTGTTGACGCCT
Nanog	CTCCAACATCCTGAACCTCAGC	CGTCACACCATTGCTATTCTTCG
4-Oct	CCTGAAGCAGAAGAGGATCACC	AAAGCGGCAGATGGTCGTTTGG
Sox2	GCTACAGCATGATGCAGGACCA	TCTGCGAGCTGGTCATGGAGTT

### Western blot analysis

Nuclear and membrane proteins were extracted from cells using the Nuclear Protein Extraction Kit (Keygenbio, Nanjing, China) and Membrane Protein Extraction Kit (Keygenbio, Nanjing, China), respectively, following the manufacturer’s instructions. Lysates were quantified spectrophotometrically using nanodrop2000 (Thermo). Lysates were separated on SDS-PAGE gels, and the proteins were transferred to polyvinylidene difluoride (PVDF) membranes (Millipore, Billerica, MA, USA). The membranes were incubated at 4°C overnight with one of the following primary antibodies: affinity purified rabbit monoclonal anti-RGS6 (1:800; Abcam Inc., Cambridge, MA, USA); anti-DNMT1 (1:1000; Abcam); anti-Nanog (1:500; Abcam); anti-Oct4 (1:500; CST Co. Inc., USA); a Na,K-ATPase antibody (1:1000, CST Co. Inc., USA), or a rabbit anti-human anti-Lamin A antibody (1:1000; Abcam). The membranes were then rinsed with PBS and incubated with a horseradish peroxidase-conjugated anti-rabbit antibody (1:3000; Sizhengbo). The blots were visualized using ECL reagent (BioRad, USA). Image acquisition and analysis of band density were performed using a G:BOX (Synoptics Group, England, UK) chemiluminescence imager and GeneSys automatic control software (10.17504/protocols.io.h4pb8vn).

### Statistical analysis

All values are given as mean ± standard deviation. Comparison between groups was carried out using SPSS version 11.0 software (SPSS Inc., Chicago, USA). A P value <0.05 was considered statistically significant.

## Results

### Predicting the HA117-RGS6 interaction and associated binding sites

Prediction of HA117-RGS6 interactions and binding sites was performed using LncTar. We selected ten confirmed splice variants as the “Target” and set HA117 as the “Query”. The cutoff value was set at -0.1. An ndG value ≤ -0.1 indicated an interaction between HA117 and alternatively spliced transcript variants of RGS6. Based on data from our experiments, we obtained ndG values of -0.096235294 and -0.084588235, suggesting two interactions and binding positions (Table 2). Co-analysis using information about functional mRNA sequences obtained from the NCBI database revealed that one of the predicted binding sites (796 to 1108 bp on the target mRNA) spanned the upstream and functional areas of the GGL domain. In this paper, we decided to focus on the interaction between HA117 and the GGL domain.

**Table 2:** Predicted HA117-RGS6 interactions.

Basic information		Cutoff: -0.1	Query		Target	
Query	Target	ndG	Start	End	Start	End
HA117	NM_001204416.1	-0.096235294	1	340	769	1108
HA117	NM_004296.5	-0.096235294	1	340	769	1108
HA117	NM_001204417.1	-0.084588235	40	340	1	301
HA117	NM_001204418.1	-0.084588235	40	340	1	301
HA117	NM_001204419.1	-0.096235294	1	340	769	1108
HA117	NM_001204420.1	-0.096235294	1	340	769	1108
HA117	NM_001204421.1	-0.084588235	40	340	1	301
HA117	NM_001204422.1	-0.084588235	40	340	1	301
HA117	NM_001204423.1	-0.096235294	1	340	769	1108
HA117	NM_001204424.1	-0.096235294	1	340	769	1108

### RNAi and RNA overexpression-mediated inhibition and up-regulation of HA117 in HL60 cells

**Fig 1:**
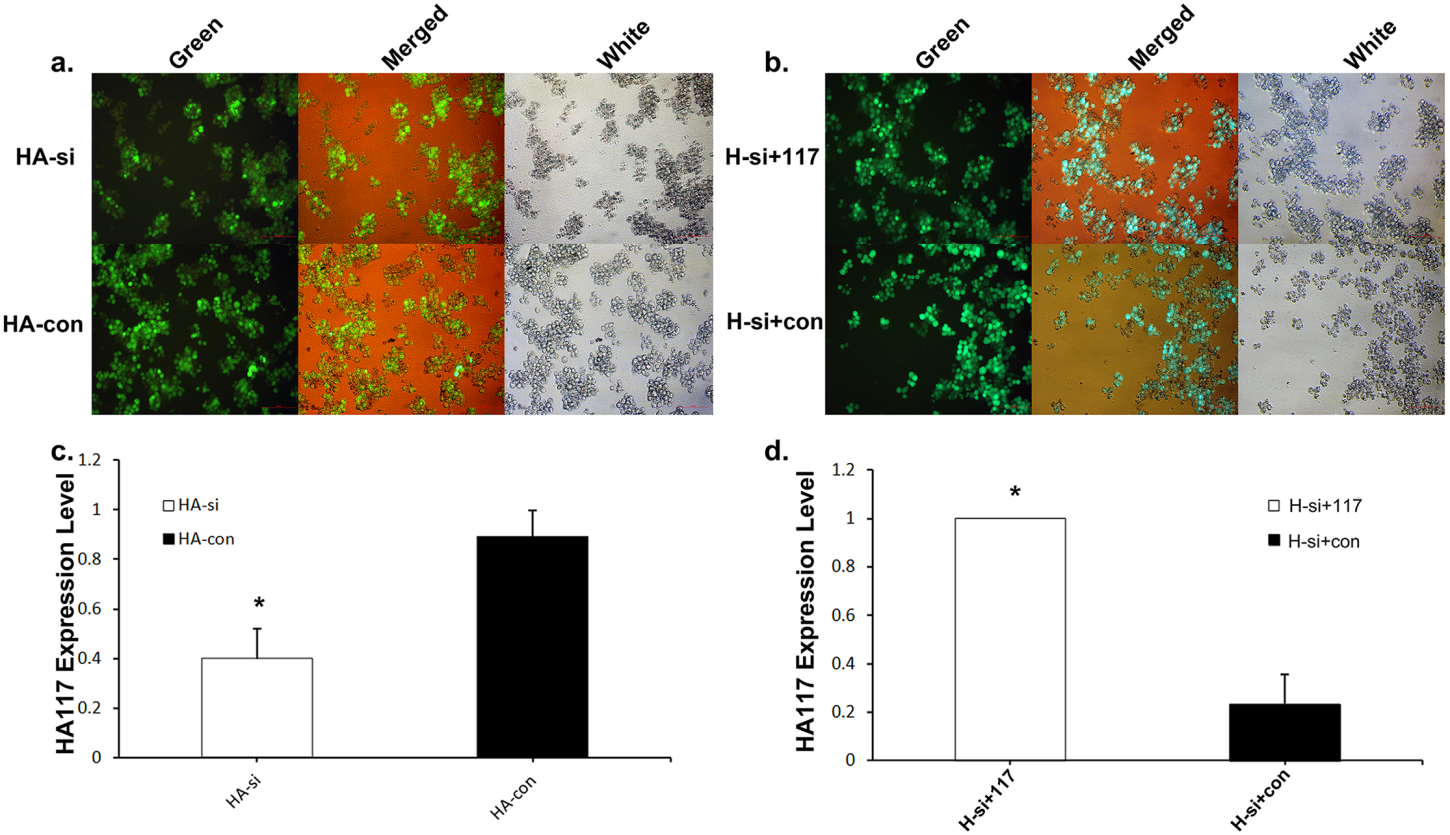
RNAi-mediated inhibition or overexpression of HA117 up-regulates HA117 expression in HL60 cells. (a) HL60 cells transfected with HA117-specific shRNA or an empty lentivirus (images acquired under light and fluorescent microscopes). (b) HA-si cells transfected with HA117 overexpression lentivirus or empty lentivirus (images acquired under light and fluorescent microscopes). Real-time PCR analysis revealed that the expression of HA117 in HA-si and HA-con cells was considerably reduced after shRNA treatment compared with that in control cells. (d) Real-time PCR analysis revealed that expression of HA117 in HA-si+117 cells was significantly higher than that in HA-si-con cells after transfection with the HA117-overexpressing lentivirus.

We used shRNA to knockdown expression of HA117 in HL60/ATRA cells (named HA-si cells) and then used an RNA-overexpressing lentivirus to rescue HA117 expression in HA117-inhibited HL60/ATRA HA-si cells (named HA-si+117 cells). The shRNA lentivirus and RNA-overexpressing lentivirus contained a GFP reporter gene and an anti-Hygromycin B (anti-HB) resistance cassette, respectively (Fig 1a and 1b). Transfected cells were enriched by picking out those resistant to HB, and HA117 RNA expression was tracked by real-time PCR (Fig 1c and 1d and S1 Dataset).

### Down-regulation or up-regulation of HA117 affects the proliferation and migration of HL60 cells

**Fig 2:**
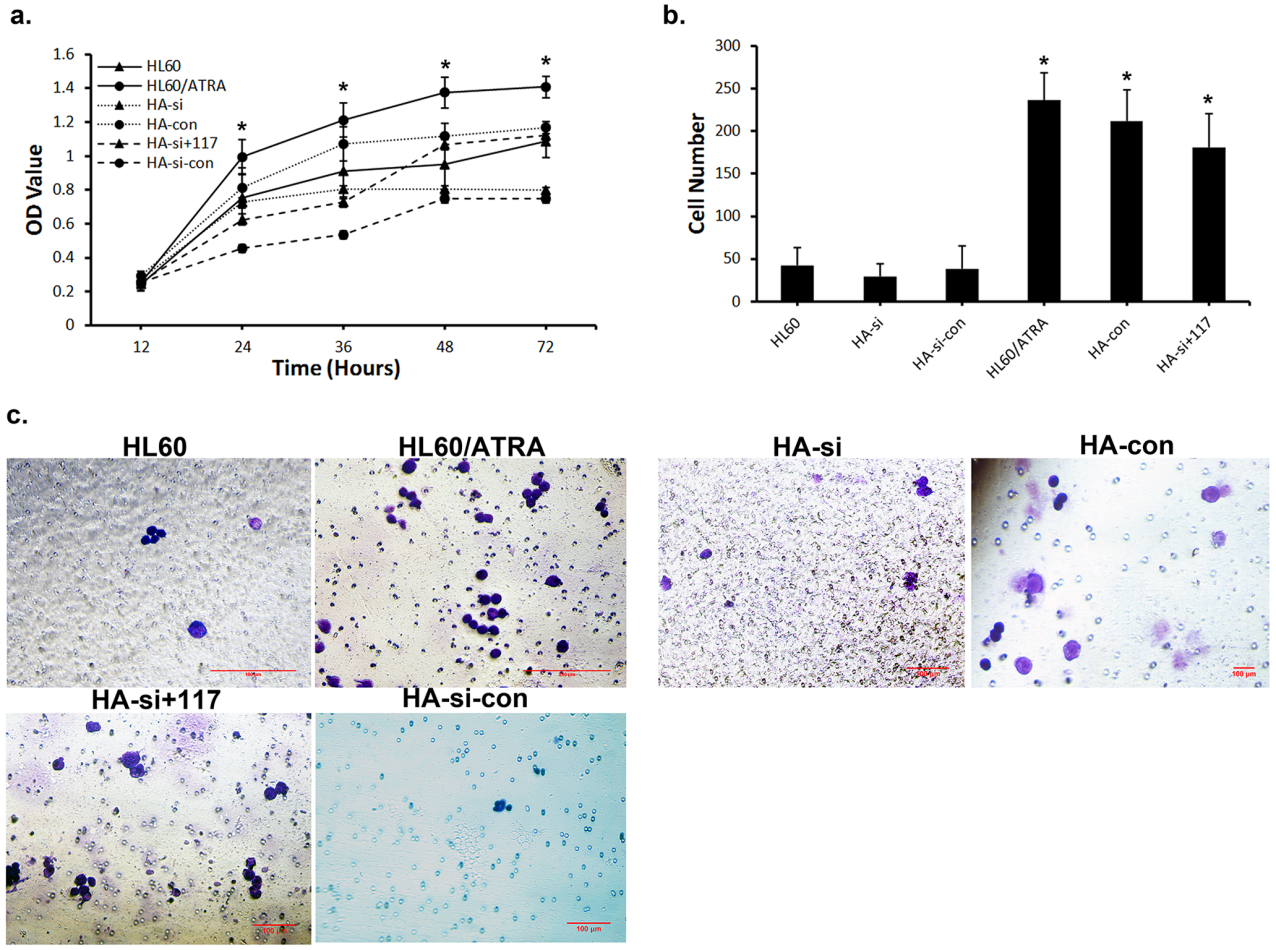
Effects of down-regulating or up-regulating HA117 expression on the proliferation and migration of HL60 cells. (a) The CCK8 assays showed that up-regulating or down-regulating HA117 expression impacted cell growth in a time-dependent manner. Data are expressed as the mean ± SD of three independent experiments (*P < 0.05). HA117 expression or its down-regulation impacted cell growth in a time-dependent manner. (b) The number of migrating cancer cells was counted in three separate fields per experimental group. Data are expressed as the mean ± SD. (*P < 0.05; ANOVA). (c) The migration of cells showing low expression of HA117 was significantly lower than that of HA117-overexpressing cells.

The cell proliferation assay revealed that wild-type HL60, HA-si, and HA-si-con cells, which showed low HA117 expression, grew considerably more slowly than HL60/ATRA, HA-con, or HA-si+117 cells (Fig 2a). Moreover, the migratory ability of wild-type HL60, HA-si, and HA-si-con cells was significantly lower than that of HL60/ATRA, HA-con, or HA-si+117 cells (Fig 2b and 2c).

### HA117 down-regulates expression of the GGL domain of RGS6

**Fig 3:**
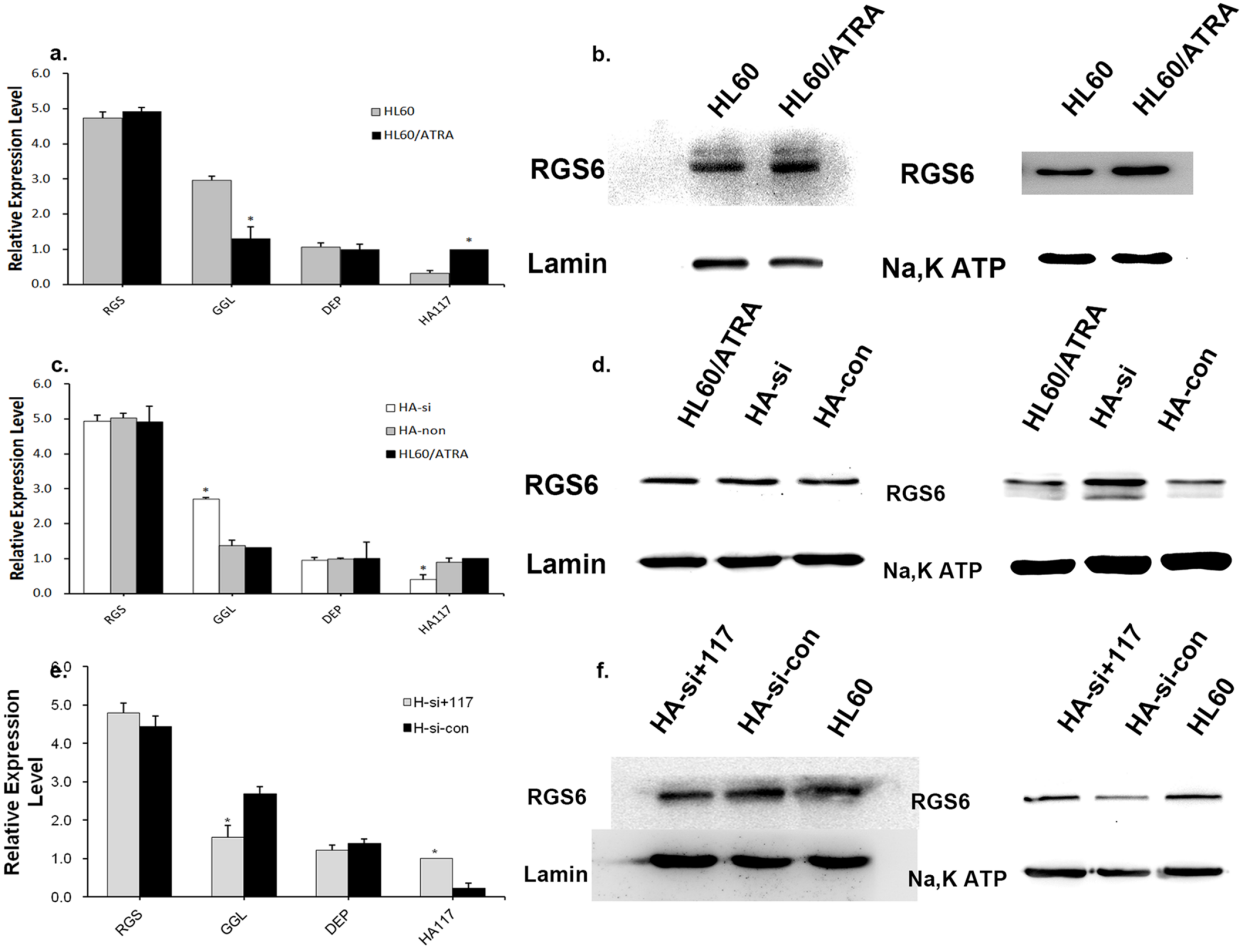
HA117 down-regulated the expression of the GGL domain of RGS6. HL60/ATRA and generation-matched wild HL60 cells were cultured in growth medium (RPIM1640 with 10% FBS, CTR) and harvested during logarithmic growth phase. Cells were then subjected to (a) real-time PCR analysis and (b) western blot analysis. HL60/ATRA cells were infected with empty lentiviral vectors (HA-con) or HA117-specific shRNAs (HA-si), and the infected cells were selected with puromycin for 2 days. These cells were amplified and harvested, and subjected to (c) western blot analysis and (d) real-time PCR analysis. Transfection of HA117 shRNA up-regulated GGL domain expression in HA-si cells. HA-si cells were infected with lentiviral vectors carrying control scrambled (HA-si-con) or full-length HA117 (HA-si+117), followed by puromycin selection of the infected cells for 2 days. Selected cells were then expanded and harvested, and subjected to (e) western blot analysis and (f) real-time PCR analysis. Transfection of full-length HA117 suppressed GGL domain expression in HA-si+117 cells.*P<0.005 (Student’s t-test). All statistical analyses were performed with Microsoft Office Excel.

To examine whether differential expression of the GGL domain accompanies overexpression of HA117, we collected generation-matched wild-type HL60 cells and drug-resistant HL60/ATRA cells and measured expression of HA117, the RGS domain, the DEP domain, and the GGL domain at the mRNA level. We observed significantly lower expression of the GGL domain in HL60/ATRA cells than in wild-type HL60 cells. There was no significant difference in expression of the RGS and DEP domains between the two cell lines (Fig 3a and S1 Dataset). The western blot results show markedly higher expression of RGS6 in the membrane protein fraction from HL60 cells than in that from HL60/ATRA cells; however, there was no significant difference in expression of RGS6 in the nuclear protein fractions from the two cell lines(Fig 3b and S2 Dataset). To confirm whether reduced expression of the GGL domain was caused by HA117, we transfected HL60/ATRA cells with the HA117 shRNA lentivirus (to generate the HA-si cell line) and used HL60/ATRA cells as the blank group. HL60/ATRA cells transfected with empty lentivirus were used as the control group (HA-non cells). The expression of HA117, RGS domain, and GGL domain mRNA was then examined in HL60/ATRA cells, HA-non cells, and HA-si cells; the expression of RGS6 was examined at the nuclear protein and membrane protein levels. We noted significantly higher expression of GGL domain mRNA in HA-si cells than in HL60/ATRA cells; however, there was no significant difference between HL60/ATRA cells and HA-non cells (Fig 3c and S1 Dataset). Expression of RGS6 in the membrane protein fraction from HA-si cells was significantly higher than in that from HL60/ATRA and HA-con cells (there was no significant difference between HL60/ATRA and HA-con). However, there was no significant difference in expression of RGS6 in the nuclear protein fractions from the three cell lines (Fig 3d and S2 Dataset). Next, we transfected HA-si cells with an HA117-overexpressing lentivirus (to yield HA-si+117 cells). HA-si cells transfected with an empty lentivirus were used as the control group (HA-si-non cells). Expression of HA117, RGS domain, and GGL domain mRNA, and RGS6 protein, was then examined. Expression of GGL domain mRNA in HA-si+117 cells was significantly lower than that in HA-si-non cells (Fig 3e and S1 Dataset), whereas expression of RGS6 protein in the membrane protein fraction from HA-si+117 and HL60/ATRA was significantly lower than in that from HA-si-con; however, there was no significant difference in expression of RGS6 in the nuclear protein fraction from the three cell lines (Fig 3f and S2 Dataset). Taken together, these results suggest that expression of HA117 down-regulates expression of the GGL domain of RGS6 and affects the cellular localization of RGS6.

### Up-regulation or down-regulation of HA117 affects drug resistance

To determine whether the drug resistance of HL60 cells is affected by HA117 expression, HL60 cells, HL60/ATRA cells, HA-si cells, and HA-si+117 cells were treated with VCR, ADM, Vp-16, DNR, MMC, or CTX for 24 h, after which cell survival/proliferation was assessed. A significant fold change in the concentration of chemotherapeutic drug required to inhibit cell proliferation by 50% (IC50) was observed in all groups (Table 3). A significant reduction in the IC50 was also observed for HA-si cells. Compared with HL60/ATRA cells, we noted a 3.16-fold, 2.99-fold, 1.81-fold, 16.15-fold, 1.95-fold, and 3.57-fold reduction in the concentrations of VCR, ADM, Vp-16, DNR, MMC, and CTX, respectively, required to inhibit the growth of HA-si cells by 50%. A significant increase in the IC50 was observed for HA-si+117 cells, with 5.78-fold, 2.34-fold, 1.86-fold, 11.08-fold, 2.62-fold, and 4.76-fold increases in the concentrations of VCR, ADM, Vp-16, DNR, MMC, and CTX, respectively, required to obtain a 50% inhibition compared with the respective values for HL60 cells.

**Table 3:** Fold resistance values for vincristine, arabinoside, doxorubicin, daunorubicin, and Cytoxan.

Drug	Half maximal inhibitory concentration (IC50)			
	HL-60	HL-60/ATRA	HA-si	HA-si+117
VCR	0.213±0.018	1.012±0.041	0.320±0.013	1.851±0.30
ADM	0.062±0.009	0.410±0.060	0.137±0.083	0.320±0.011
Vp-16	4.209±0.018	12.732±0.780	7.021±0.104	13.031±0.102
DNR	0.084±0.012	0.856±0.095	0.053±0.002	0.587±0.032
MMC	3.677±0.349	11.237±1.062	5.756±0.887	15.102±0.652
CTX	1.897±0.138	6.920±0.211	1.937±0.121	9.221±0.200

### HA117 inhibits DNMT1 degradation by down-regulating expression of the GGL domain

**Fig 4:**
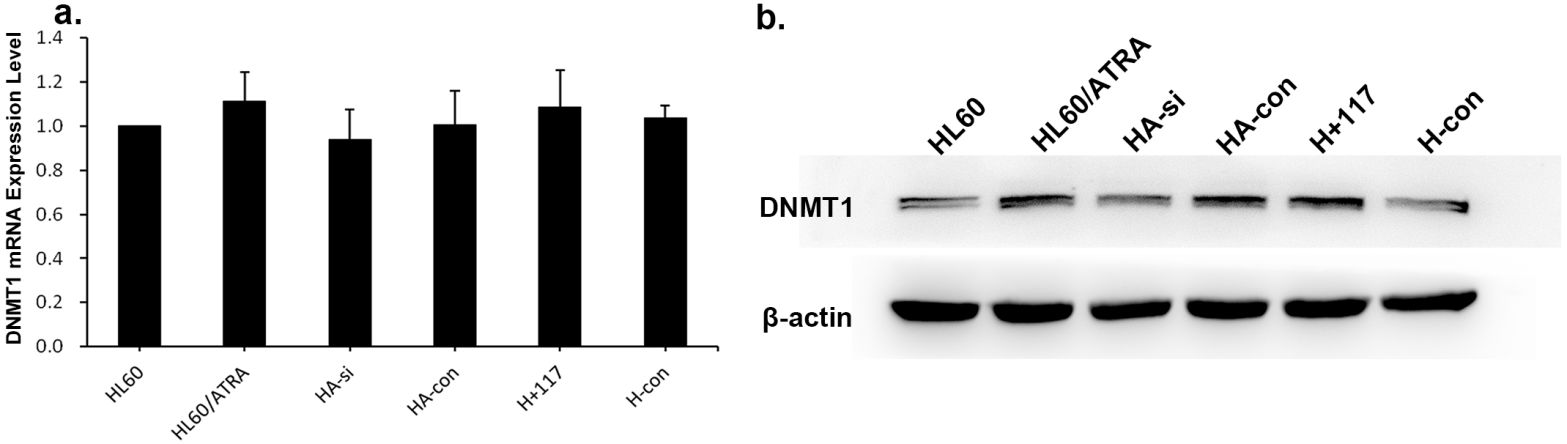
Chemoresistant cell lines showed increased DNMT1 expression. DNMT1 mRNA (a) and protein (b) expression was examined in all cell lines. HL60/ATRA, HA-con, and HA-si+117 cells showed higher protein but not RNA expression of DNMT1 than HL60, HA-si, or HA-si-con cells. Data are expressed as the mean ± SEM of five independent experiments (*p ≤ 0.05).

To investigate the expression of DNMT1 in HA117-expressing cell lines, and to examine whether it is affected by down-regulation of the GGL domain, we measured expression of HA117, GGL domain, and DNMT1 mRNA and DNMT1 protein. We found no significant difference in DNMT1 mRNA expression in any of the cell lines (Fig 4a and S1 Dataset). By contrast, DNMT1 protein expression was significantly increased in HL60/ATRA cells and in cells overexpressing HA117 (Fig 4b and S2 Dataset).

### Drug-resistant cell lines exhibit increased expression of cancer stem cell markers

**Fig 5:**
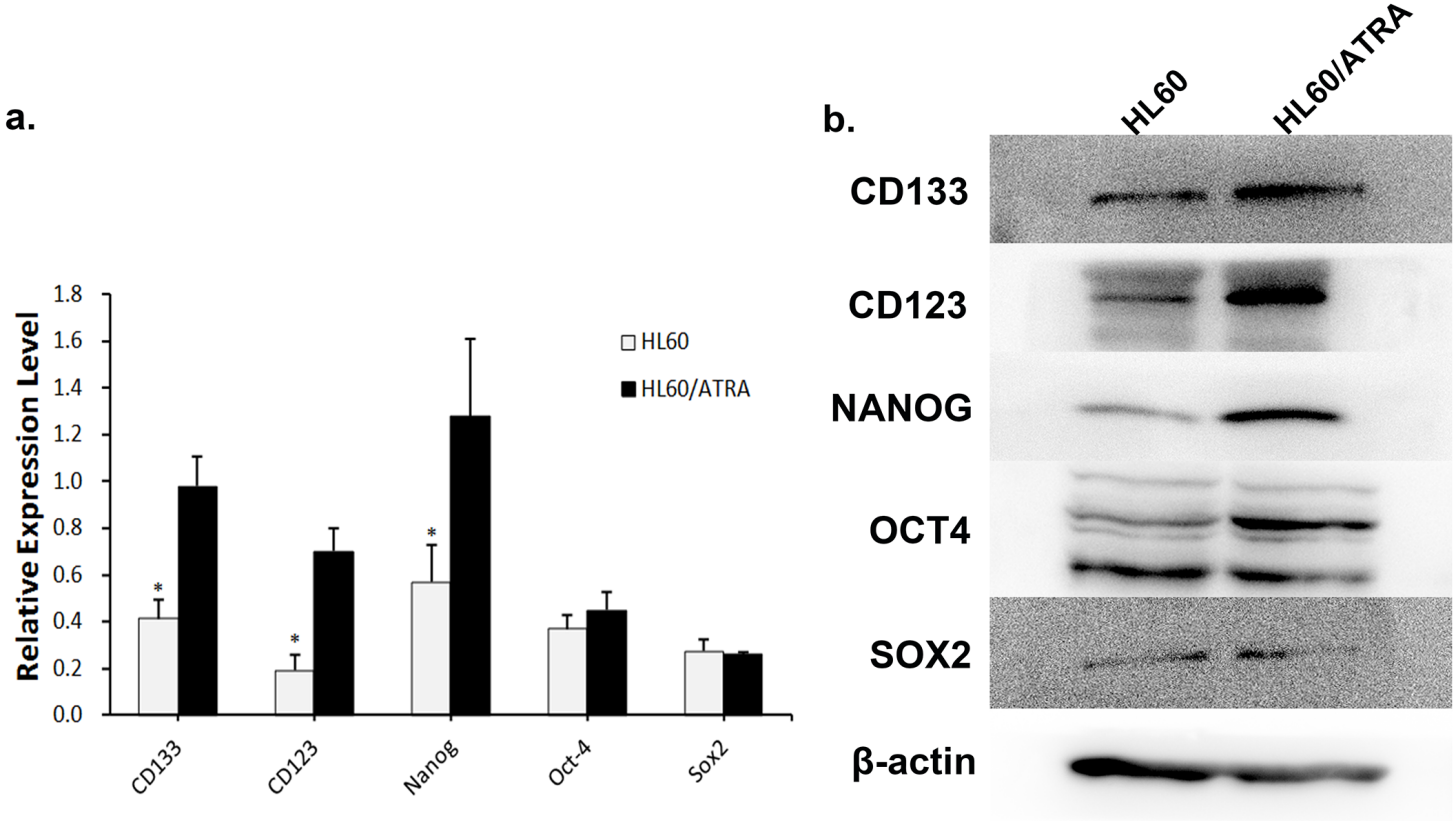
Drug-resistant cells show increased expression of cancer stem cell markers. Total RNA and total protein were isolated from wild-type HL60, HL60/ATRA cell lines and subjected to real-time PCR (a) and SDS-PAGE gel electrophoresis/western blot analysis (b). Expression of Nanog, Oct-4, SOX-2, CD133 and CD123 mRNA and protein was then examined. HL60/ATRA cells showed higher expression of CD133, CD123, and Nanog than HL60 cells. Data are expressed as the mean ± SEM from five independent experiments (*p, 0.05).

Previous studies show that up-regulating DNMT1 increases expression of cell stem-like markers [17, 18]. To examine expression of cancer stem cell markers by the drug-resistant cell lines, we screened them for a number of key human embryonic or cancer stem cell markers at both the mRNA and protein level. Embryonic stem cell markers Nanog, Oct-4, and SOX-2, the cancer stem cell marker CD133, and the leukemia stem cell marker (CD123) [19] were examined (Fig 5, S1 and S2 Dataset). We observed differential expression of markers between drug-resistant and wild-type cell lines. The former showed higher expression of Nanog than parental cells. Expression of CD133 and CD123 was also significantly higher in the drug-resistant lines. However, there was no significant difference in the expression of Oct-4 or SOX-2 between drug-resistant and wild-type cell lines.

## Discussion

Since its introduction in clinical trials, ATRA has had a major effect on the therapy of Kaposi’s sarcoma, APL, neck squamous cell carcinoma, head and ovarian carcinoma, bladder cancer, and neuroblastoma [20]. To date, ATRA-based combinations are the most effective systemic chemotherapy for promyelocytic leukemia and remain the standard first-line chemotherapy for APL. ATRA, however, poses a number of major problems, one of which is the acquisition of drug resistance, which undermines its curative potential. Identification of the mechanism underlying the drug-resistant phenotype associated with ATRA-induced resistance is crucial for successful clinical cancer treatment. An example highlighting this limitation is neuroblastoma patients who experience more than 50% tumor recurrence, primarily because the tumors become resistant to therapy [21]. Similarly, in APL the relapse rate can be 10–30% [22, 23]. A further therapeutic complication is that tumors that fail to respond to ATRA develop cross-resistance to other unrelated anti-tumor drugs [24]. This indicates that ATRA and other agents might share common mechanisms of resistance. There is an pressing need to better understand the molecular mechanisms underlying the ATRA-resistant phenotype, since ATRA-based chemotherapy for some tumors appears to have arrived at a plateau in efficacy[25, 26].

In a previous study, we generated an isogenic model of ATRA-induced multi-drug resistance using a panel of HL60 cell lines derived from original, generation-matched parent cell lines and then characterized these in terms of their proliferative and apoptotic potential and cell cycle distribution. Chronic exposure to increasing concentrations of ATRA led to the development of multi-drug-resistant cell lines. The IC50 values of the ATRA-treated cell lines were significantly higher than those of the parental cells, demonstrating acquisition of a more resistant phenotype. Furthermore, we identified a new gene fragment, named HA117, in ATRA-induced drug-resistant HL60/ATRA cells [3]. Expression of HA117 in cell lines and clinical samples is associated with drug resistance and patient prognosis [27–29].

Here, we demonstrated that HA117 induces HL60/ATRA cells to express a stem-like multi-drug resistance signature by inhibiting the degradation of DNMT1 via down-regulation of the GGL domain of RGS6. One of the most noticeable cellular responses to HA117 overexpression was down-regulation of the GGL domain of RGS6, which plays an important role in DNMT1 degradation. The LncTar tool was used to detect possible interactions between HA117 and RGS6; furthermore, the binding regions of those two genes were identified within the 769 to 1108 bp region of the RGS6 sequence, which is located upstream and the full sequence of GGL domain of RGS6. Exon skipping is a common method of alternative splicing that is always induced by a specific antisense oligonucleotide [30]. To find out whether HA117 causes alternative splicing of RGS6 by binding to the RGS6 sequence, we used qPCR to examine expression of the GGL domain while HA117 was silenced or overexpressed at the mRNA level; expression of RGS6 protein was examined by western blotting. We found that expression of the GGL domain was low when HA117 was overexpressed, whereas GGL domain expression was restored when HA117 was silenced. Moreover, expression of the GGL domain affects the cellular localization of RGS6 [7]; we found that, when HA117 was overexpressed, expression of RGS6 in the membrane protein fraction was significantly lower than when HA117 expression was low. These data suggest that expression of HA117 affects expression of the GGL domain. DNMT1 is one of several genes responsible for maintaining methylation patterns following DNA replication; some studies show that activation of DNMT1 maintains stem cells [13, 14, 17, 31]. However, RGS6 can function as a scaffold protein for both Tip60 and DNMT1. Tip60 binds directly or indirectly to the RGS domain of RGS6 located adjacent to the GGL domain, which Dnmt1 binds to via DMAP1, and this interaction is required for Tip60-mediated acetylation of DNMT1 and its subsequent ubiquitination and degradation[9]. Here, we found that, while expression of HA117 might not affect that of DNMT1 at the mRNA level, overexpression suppresses expression of DNMT1 protein; this is reversed when HA117 is silenced. These data suggest a possible link between HA117 expression and epigenetic regulation of cancer stem cell signatures.

Another noticeable response to HA117 overexpression in HL60 cells was the endowment of a stem-like signature. We found that multi-drug-resistant HL60/ATRA cells showed higher expression of the cancer stem cell marker CD133 [32] and the leukemia stem cell marker CD123 [19] than wild-type HL60 cells. Evidence that ATRA-induced drug-resistant subpopulations of cells within our cell lines had characteristics consistent with a putative cancer stem-like signature was further supported by screening cells for the expression of several other embryonic stem cell markers. For example, resistant HL60/ATRA cells showed higher expression of Nanog than wild-type HL60 cells. However, while this increase in Nanog expression suggested a pluripotency regulation network, a significant increase in SOX-2 protein and Oct-4 expression in HL60/ATRA cells was not observed. Recent studies have shown that cancer stem cells are more tumorigenic than differentiated cancer cells, and that the transcription factor Nanog plays a crucial role in maintaining the unique properties of stem cells [33, 34]. Cao et al. showed that knockdown of Nanog in leukemic cells inhibits cell proliferation, reduces self-renewal, and arrests the cell cycle [35]. Han et al., also showed that Nanog regulates the migration and proliferation of cancer cells [36]. Our ex vivo experiments showed that the up-regulation of HA117 expression increased migration and cancer cell proliferation. These results provide evidence that HA117-mediated up-regulation of Nanog regulates the migration and proliferation of cancer stem-like cells to promote the development of human cancer.

## Conclusions

In conclusion, we observed down-regulated expression of the GGL domain of RGS6 and up-regulation of DNMT1 in a panel of ATRA-induced multi-drug-resistant HL60/ATRA cells. Both the presence and enrichment of stem cell markers supports the presence of a chemoresistant population of HL60/ATRA cells with a stem-like signature; these cells may be clinically relevant for studying the mechanisms of ATRA-induced multi-drug resistance in an ex vivo model. We have shown that HA117 induces a stem-like signature in drug-resistant HL60/ATRA cells by inhibiting the degradation of DNMT1 via down-regulated expression of the GGL domain of RGS6. Overexpression of HA117 up-regulated DNMT1 expression and subsequent expression of cancer stem cell markers. These data suggest that targeting HA117 and DNMT1 could constitute a new strategy for treating drug-resistant cancers.

## Supporting information

S1 Dataset All qPCR raw datas. (RAR)

S2 Dataset All Western Blot raw datas. (RAR)

## Abbreviations

ADM: doxorubicin
ATRA: all-trans retinoic acid
CCK8: Cell Counting Kit-8
CD123: Interleukin-3 receptor alpha chain
CTX: cyclophosphamide
DNMT1: DNA methyltransferase 1
DNR: daunorubicin
GFP: green fluorescent protein
GGL domain: G-protein gamma sub-unit-like domain
MMC: mitomycin C
mRNA: messenger RNA
RGS6: G-protein signaling 6
RNAi: RNA interference
shRNA: short hairpin RNA
VCR: vincristine
Vp-16: etoposide phosphate
